# Freeze Casting of Microporous Composite Beads Based on a Polymer of Intrinsic Microporosity for Gas Storage Applications

**DOI:** 10.1021/acsomega.4c07247

**Published:** 2025-04-17

**Authors:** Catherine Butler, Bastola Narayan, Timothy J. Mays, Tristan Lowe, Rachel O’Malley, Vijay Sahadevan, Christopher R. Bowen

**Affiliations:** † Department of Mechanical Engineering, University of Bath, Claverton Down, Bath BA2 7AY, U.K.; ‡ Department of Chemical Engineering, University of Bath, Claverton Down, Bath BA2 7AY, U.K.; § Henry Moseley X-ray Imaging Facility, Photon Science Institute, The University of Manchester, Alan Turing Building, Oxford Road, Manchester M13 9PL, U.K.; ∥ Global Technology Centre, GKN Aerospace, Taurus Road, Patchway, Filton, Bristol BS34 6FB, U.K.

## Abstract

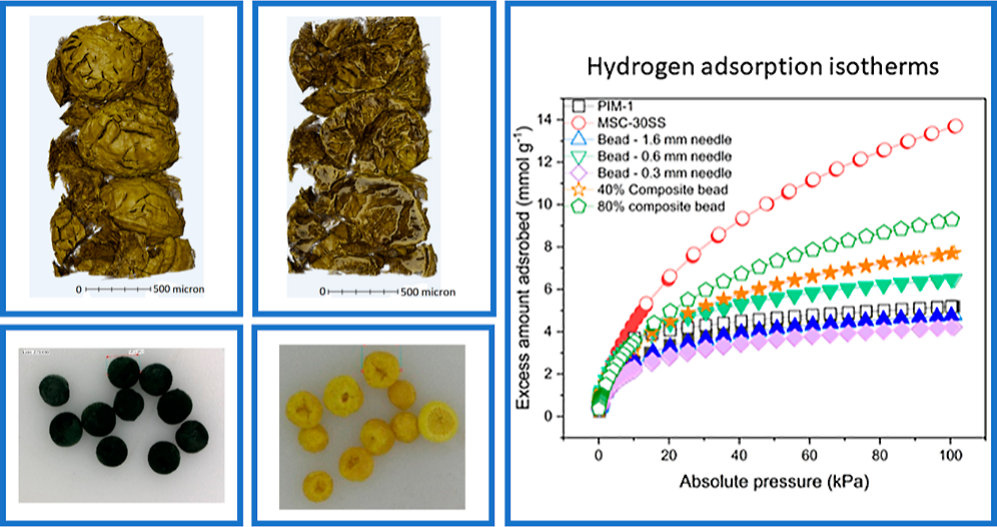

Polymers of intrinsic microporosity (PIMs) show potential for gas storage and separation applications. However, they are often produced in fine-scale particulate form which can lead to handling and fouling issues, for example when inserted inside storage tanks. We provide the first demonstration of a manufacturing technique to form millimeter scale PIM-based beads by freeze casting PIM-1 droplets. The PIM-based beads are shown to exhibit similar Brunauer–Emmett–Teller (BET) surface areas compared to its original particulate form and low pressure hydrogen isotherms (to 0.1 MPa) are presented to examine gas storage behavior. We also demonstrate the production of composite PIM-1 beads containing up to 80 wt % of activated carbon filler, which leads to improved surface area for gas storage and separation, generally following the rule of mixtures. The composite beads can store 1.6 wt % hydrogen at 0.1 MPa and 77 K and are predicted to have a maximum storage capacity of 8.1 wt %, with potential to meet the Department of Energy (DoE) targets for light duty fuel cell vehicles.

## Introduction

**Figure 1:**
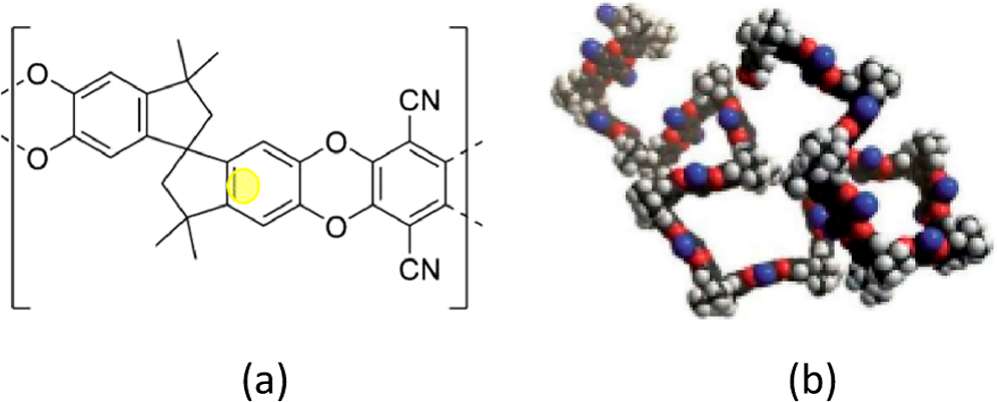
(a) Structure of PIM-1 showing spiro-center (yellow circle) which limits packing of polymer chains and creates pores, many in the micropore size range (b) physical representation of structure.

Polymers of intrinsic microporosity (PIMs) were introduced by Budd et al. in 2004^1^ as a novel new class of glassy polymer that contain intrinsic porosity of dimensions less than 2 nm, namely in the micropore size range. The PIM-1 polymer has a rigid, but kinked structure, and rotation is restricted around the polymer backbone and spiro-centers, sites of contortion, as highlighted in Figure 1. This inhibits efficient space packing of the polymer, thereby creating pores (many in the micropore size range) (<2 nm). The porosity is described as intrinsic since it results from the monomer structure, as opposed to being introduced by material processing.^2^ The nitrogen isotherm for PIM-1 at 77 K demonstrates type IV behavior which indicates the presence of mesopores as well as micropores.^1^ PIM-1 shows potential to be processed into a range of morphologies, while being able to retain its porosity and exhibit good thermal and mechanical properties. This combination of properties and structure has made it a material of interest for applications related to gas storage,^3^ including for hydrogen.

The hydrogen storage capacity of a material is linked to the accessible surface area. In this regard, PIM-1 has exhibited a relatively limited surface area (724 m^2^ g^–1^ ^4^) from nitrogen sorption experiments at 77 K. However, it exhibits a capacity to incorporate high surface area fillers to create composite systems which can enhance the overall surface area and adsorption capability. Recent publications, summarized below, have focused on forming storage materials into useable geometries with appropriate hydrogen storage, mechanical, and thermal properties to withstand tank conditions; however, they are not without challenges. Attempts to form geometries of adsorbent materials include the production on zeolitic imidazolate framework (ZIF-8) pellets using twin screw extrusion.^5^ The excess amount of hydrogen adsorbed showed a maximum value of 2.8 wt % which is not sufficient to reach the Department of Energy (DoE) targets for light duty fuel cell vehicles. In addition, pellets are not easily scalable in size. In the work of Romanos et al.^6^ an activated carbon (MSC-30) was formed into a pellet by compacting the powder in a die with a binder. While the pellet was formed successfully, this process is not easy to scale into larger geometries or a range of morphologies. In addition, the gravimetric energy density of the material decreases as a result of the forming method, which is clearly undesirable.

PIM-1 has been successfully formed into thin films by evaporating a solution of the material in chloroform.^7^ PIM-1 has also been formed into composite films using the same method, where the PIM-1 is doped with activated carbons or metal organic frameworks (MOFs) to improve the hydrogen storage capacity.^9^ However, the thickness of the films that can be produced is limited which inhibits their capacity for hydrogen storage and they are difficult to reproduce at scale. Zhang et al.^11^ developed a method for 3D-printing PIM-1, however they observed the formation of a skin layer, which restricts mass transfer and makes the approach unsuitable for applications which rely on processes such as adsorption. Ahmed et al.^12^ used solvent templating to produce a composite monolith of PIM-1 and a HKUST-1 MOF. However, the Young’s Modulus of the resulting monolith was only 33 kPa, much smaller than that predicted from other publications,^9^ up to 1 GPa. The BET surface area of the monolith is also similar to that of PIM-1, which means that the hydrogen storage capacity would not reach the DoE requirements for light duty fuel cell vehicles.^8,10,13^

In this work, droplet freeze casting of a solution of PIM-1 and MSC-30SS filler is employed to overcome the above challenges in processing these materials into useable geometries while also providing a microporous binder that can facilitate hydrogen adsorption. The resulting beads exhibit good gas storage performance and are easier to handle than micrometer scale powders when used for gas storage applications. The advantage of using a microporous PIM-1 as a binder is that it contributes to the storage of hydrogen. The hydrogen storage capacity of the composite beads is found to follow the rule of mixtures with respect to the weight fraction of each material. As a result, the capability of the high surface area material is retained while benefiting from the ease of forming of the polymer matrix to create useable bead geometries for application in hydrogen storage vessels.

## Experimental Section

### Synthesis of PIM-1

First, PIM-1 was prepared following a procedure published by Rochat et al.^14^ where anhydrous potassium carbonate (K_2_CO_3_) (16.59 g, 120 mmol), 3,3,3′,3′-tetramethyl-1, 1′-spirobisindane-5,5′,6,6′-tetraol (5.11 g, 14.6 mmol), and tetrafluoroterephthalonitrile (3.0 g, 14.7 mmol) were stirred in dry dimethylformamide (DMF, 100 mL) in a round bottomed flask, under 1 bar nitrogen at 65 °C for 72 h. Once cooled, the mixture was poured into a beaker of distilled water (300 mL) and filtered to collect the product as a yellow solid. This was repeated two more times with water and then a third time using acetone. The powder was then dried under vacuum before the solid was dissolved in 100 mL chloroform and pipetted into 900 mL of methanol. The powder was collected by filtration and dried. This process was repeated two more times before the PIM-1 powder was finally collected and dried under vacuum (1 × 10^–6^ MPa) at 80 °C for 6 h.

### Thermogravimetric (TGA) Analysis of PIM-1 and MSC-30SS

Thermogravimetric analysis was conducted using a Setaram Setsys Evolution 16/18. Samples were heated from room temperature to 850 °C at 10 °C min^–1^. A sample size of ∼10 mg was used for all materials. The samples were analyzed in flowing air and in argon, both with an initial 2 h argon purge.

### Bead Manufacture

**Figure 2:**
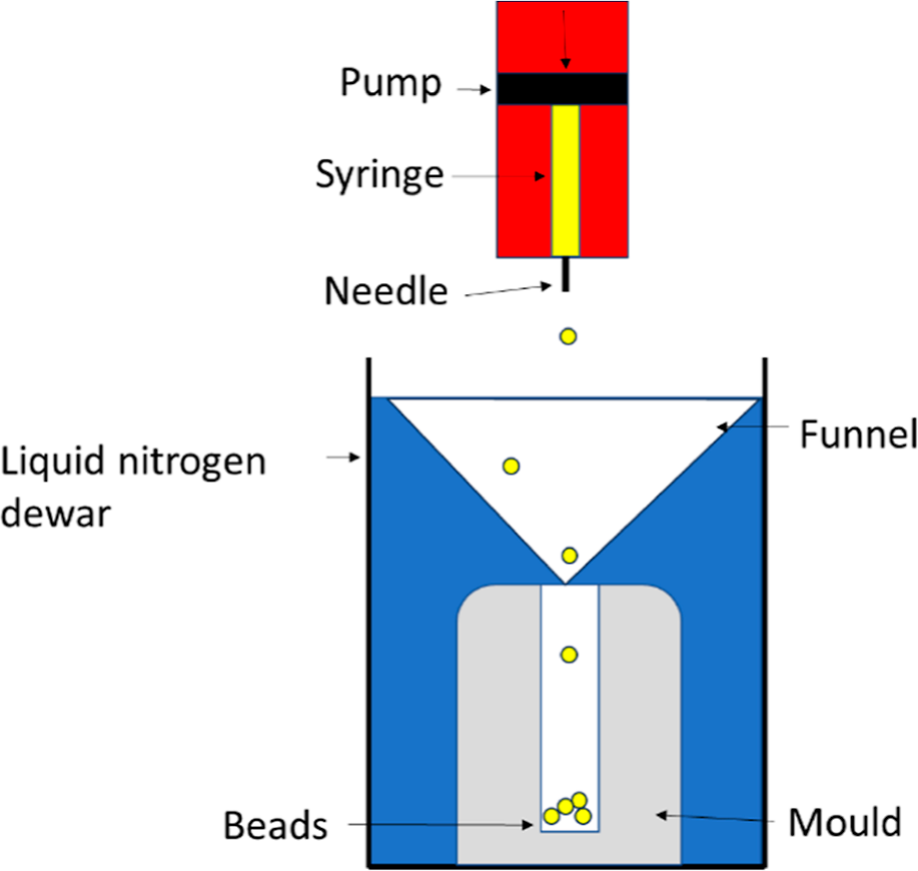
Experimental setup for bead manufacture (internal needle diameters of 0.3, 0.6 and 1.6 mm).

To manufacture PIM-1 beads, a solution of PIM-1 (0.4 g) and chloroform (5 mL) was mixed in a round bottomed flask at 20 °C and 300 rpm for 1 h. The solution was then decanted into a syringe and connected to a syringe pump (World Precision Instruments AL-300) which activated the syringe at a constant speed (60 μL h^–1^). The syringe was placed above a dewar of liquid nitrogen^15^ with a preprepared resin collection mold that was submerged in the liquid and a funnel to direct the beads into the mold for collection; see Figure 2. The syringe was connected to three different needles of internal diameter 0.3, 0.6, 1.6 mm, which were cut horizontally to release the solution evenly; this has potential to change the droplet, and the resulting bead, dimensions. The solution was dropped into the liquid nitrogen through the needles and the beads collected in the mold; see Figure 2. The mold was removed from the dewar, ensuring the beads remained submerged in liquid nitrogen, to prevent them from melting. The mold was then placed into a freeze drier (Mini Lyotrap.) and the liquid nitrogen and chloroform were then removed by sublimation, leaving the solid PIM-based beads behind.

For the 40 wt % MSC-30SS beads, 0.24 g of PIM-1 and 0.16 g of activated carbon were combined (total mass = 0.4 g, equal to the mass of PIM-1 used for the PIM-1 beads), and for the 80% carbon beads, 0.08 g PIM-1 and 0.32 g activated carbon, were mixed (total mass = 0.4 g, equal to the mass of PIM-1 used for the PIM-1 beads) in 5 mL of chloroform at 20 °C and 300 rpm for 1 h. The beads were manufactured using the same method as described for the PIM-1 beads, with a 1.6 mm internal diameter needle. Maxsorb MSC-30SS was selected as the carbon filler, which is commercially available from Kansai Coke and Chemicals Co. Ltd. in Japan. The material is an alkali activating activated carbon that has been mass produced and is synthesized from petroleum coke, mixed with excess amount of potassium hydroxide (KOH) and dehydrated at 400 °C, followed by activation at 600–900 °C in an inert atmosphere.^16^ The literature indicates it has a specific surface area of 2800–3400 m^2^ g^–1^.^16^

### Characterization of Beads: Microscopy

Microscopy of the beads was undertaken using a Keyence VHX 6000 optical microscope to examine their morphology and determine their mean diameter. The samples were also mounted in Kapton tubing and analyzed using a ZEISS Versa620 X-ray Microscope with a ×4 magnification lens. Samples were scanned at 80 keV using 125 μA with the X-ray source and detector positioned 12 and 9 mm respectively away from the center of rotation. For the X-ray CT scan 2001 radiographs were collected with each radiograph projection time being 0.5 s. The data were reconstructed using ZEISS Scout and ScanTM Control System Reconstructor—16.2.18058.47373. The reconstructed data were segmented using Reactiv’IP IPSDK 4.0 machine learning module while the segmented data was visualized using Avizo 2020.2.

### Characterization of Beads: Surface Area

Nitrogen adsorption isotherms were produced for the region *p*/*p*_0_ = 0.05–0.3^17^ on a 3Flex Instrument from Micrometrics at 77 K to determine the Brunauer–Emmett–Teller (BET) surface area of the materials. Here *p* is absolute pressure and *p*_0_ is saturation pressure. The temperature was maintained at 77 K throughout the experiment using a dewar of liquid nitrogen and an isothermal jacket. Oxygen free nitrogen (UN1066) was used, and an equilibration time of 10 s was allowed for each pressure change. The British standard (BS ISO 9277:2010) was used to estimate the BET surface area in m^2^ g^–1^. Prior to analysis, a degas process was carried out at 200 °C under vacuum (6.7 × 10^–6^ MPa) for 12 h for PIM-1 and the composite beads. For the MSC-30SS powder, the degas process was carried out at 350 °C for 8 h under vacuum (6.7 × 10^–6^ MPa) in order to remove moisture and solvents from the pores of the powder samples.

### Hydrogen Storage Capacity of Beads

Hydrogen adsorption isotherms at low pressure (up to 0.1 MPa) and 77 K were carried out on a 3Flex sieverts type instrument from Micrometrics. The temperature was maintained using a dewar of liquid nitrogen and an isothermal jacket. High purity hydrogen (BIP Plus UN1049) was used, and an equilibration time of 45 s was allowed for each pressure change. Samples with a mass of ∼100 mg were used in the analysis. Prior to analysis, a degas procedure was carried out at 200 °C under vacuum (6.7 × 10^–6^ MPa) for 12 h for the PIM-1 and PIM-1 composite beads. When testing the MSC-30SS powder the degas was carried out at 350 °C for 8 h under vacuum (6.7 × 10^–6^ MPa), to remove moisture and solvents from the pores of the sample.

Hydrogen adsorption experimental data were plotted and fitted to the Tóth isotherm (eq 1)^18^ using a Levenburg–Marquadt^19^ nonlinear curve fit in OriginPro 2024 software to predict the isotherm at higher pressures, to identify the maximum hydrogen storage capacity and determine the pressure at which this would occur. The Tóth isotherm is known to be a good model for many simple (type I) isotherms.^20^ This isotherm is given by eq 1.
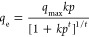
1where *q*_e_ is the measured excess uptake of hydrogen at equilibrium (mmol g^–1^), *q*_max_ is the maximum adsorbate uptake of hydrogen or capacity in mmol g^–1^, *k* is the affinity parameter in kPa^–1^, *p* is the measured absolute pressure in kPa, and *t* is a parameter that describes the surface heterogeneity of the sample. *q*_max_, *k* and *t* are adjustable parameters estimated from fitting eq 1 to the isotherm data.

## Results and Discussion

**Figure 3:**
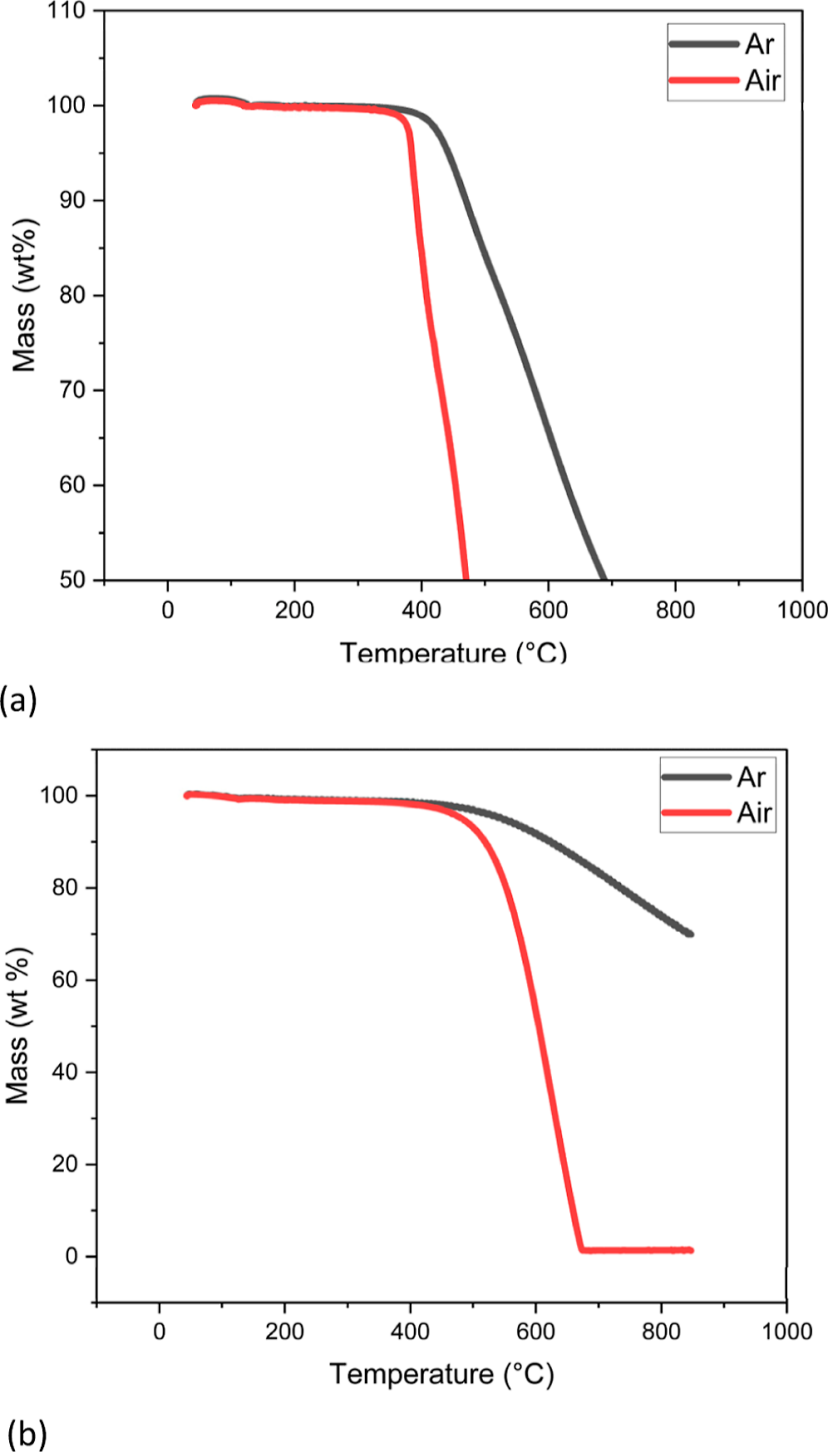
TGA analysis of (a) PIM-1 and (b) MSC-30SS in air and argon.

TGA results for PIM-1 and MSC-30SS are shown in Figure 3. The mass of PIM-1 remains constant until it reaches a temperature of approximately 400 °C, where the mass starts to decrease rapidly under air, and in argon at around 420 °C.

The MSC-30SS activated carbon begins to oxidize in air at 600 °C, with the mass remaining at the end of the analysis close to 0 mg, which indicates that there are few impurities in the sample. Under argon, the mass begins to decrease for MSC-30SS at approximately the same temperature as for air; 600 °C, which may be due to a small amount of air being present in the chamber when the analysis began. The relatively low mass change overall for the carbon under argon is small as would be expected.

**Figure 4:**
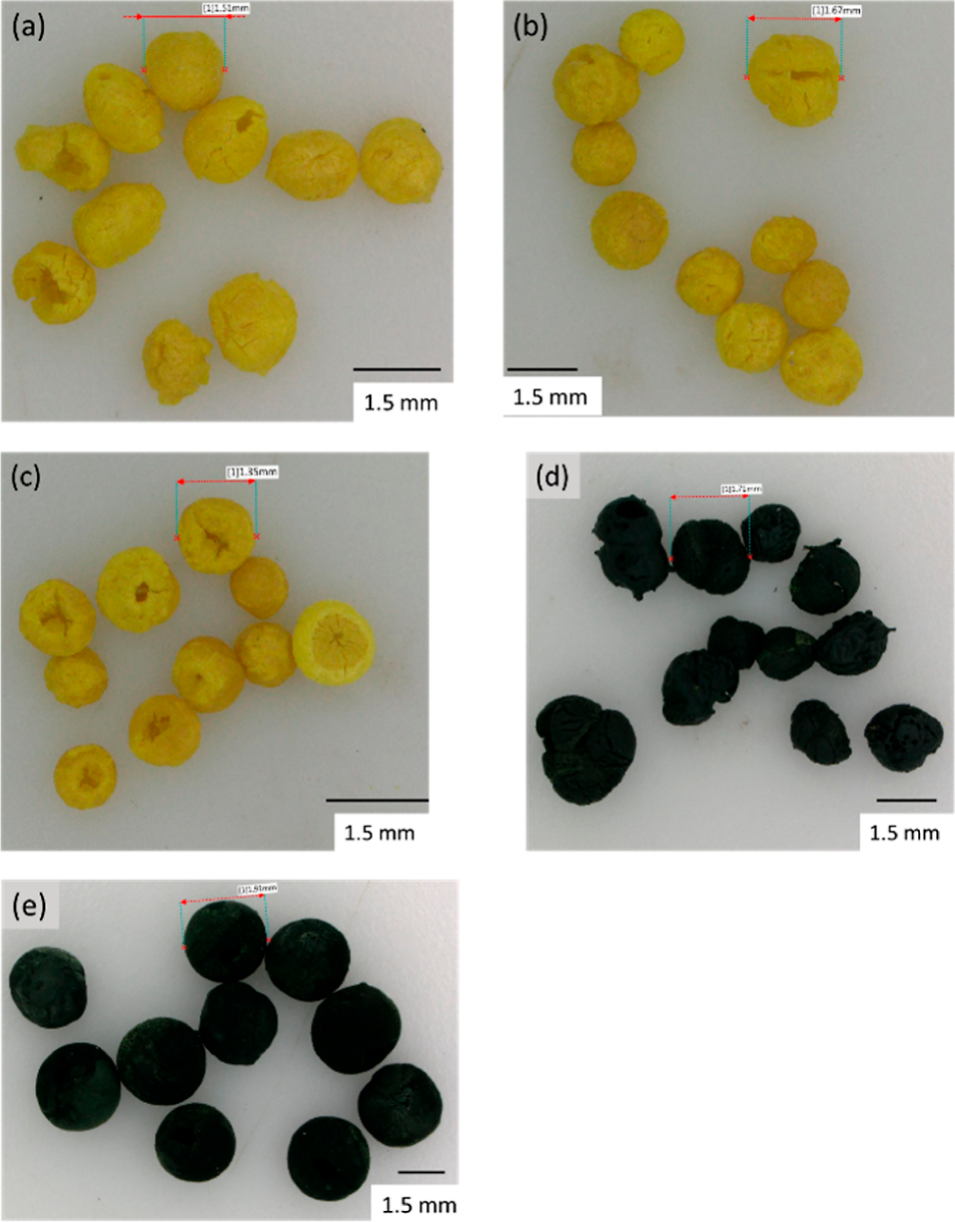
Microscopy images of beads from a (a) 1.6 mm diameter needle, (b) 0.6 mm diameter needle, (c) 0.3 mm diameter needle, (d) 40% composite beads using 1.6 mm needle, and (e) 80% composite beads using 1.6 mm needle.

Optical microscopy images of the PIM-1 beads are presented in Figure 4a–c for a range different needle sizes (0.3–1.6 mm). Figure 2d shows optical microscopy image of the 40% composite bead and 2e the 80% composite bead.

Figure 4 shows the PIM-1 beads are a relatively consistent morphology, almost spherical, where there is a general trend that a larger needle results in a larger bead. The beads are 1.5–2 mm for the 1.6 mm diameter needle, 1.2–1.7 mm for the 0.6 mm needle and 1.0–1. Three mm for the 0.3 mm needle. The (PIM-1)-activated carbon composite beads exhibit the same structure as the PIM-1 beads, with a spherical shape and there is a larger variation in size (1.2–2.3 mm for the 1.6 mm needle) of the 40 wt % MSC-30SS beads. For the 80 wt % MSC-30SS beads, also formed with the larger 1.6 mm needle, the shape and size appear larger and more uniform. The (PIM-1)-activated carbon composite beads were larger than the PIM-1 beads, and the presence of an increasing fraction of solid carbon filler may act to reduce shrinkage of the bead during the freezing and freeze-drying process, which also improves the uniformity of the resulting bead dimensions and morphology. The composite beads also appear homogeneous in color, indicating that the MSC-30SS carbon filler is evenly spread throughout the bead structure.

**Figure 5:**
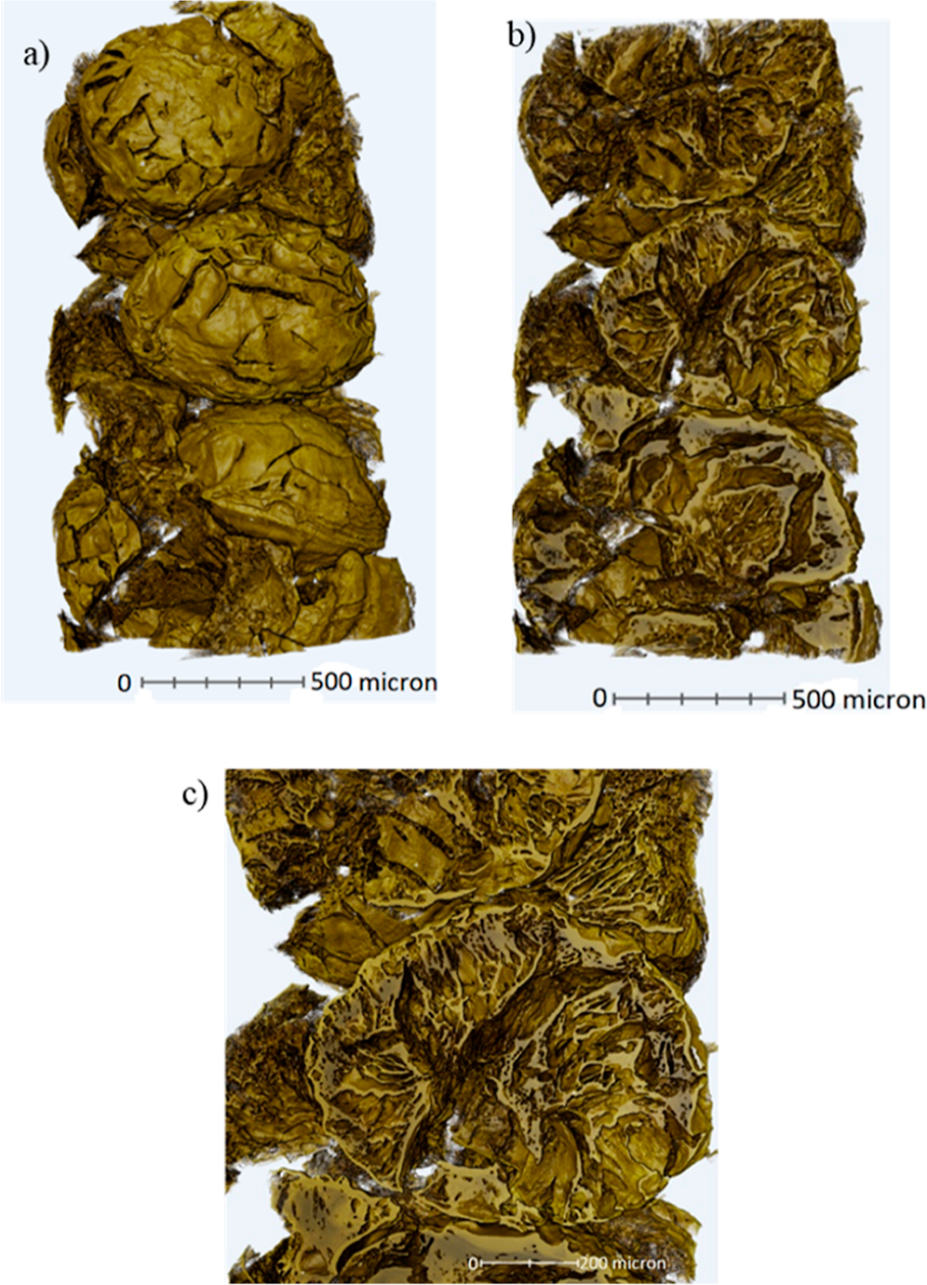
3D rendered CT scan images of 0.6 mm beads (a) outer surface, (b) section through beads, (c) higher magnification of section through beads.

**Figure 6:**
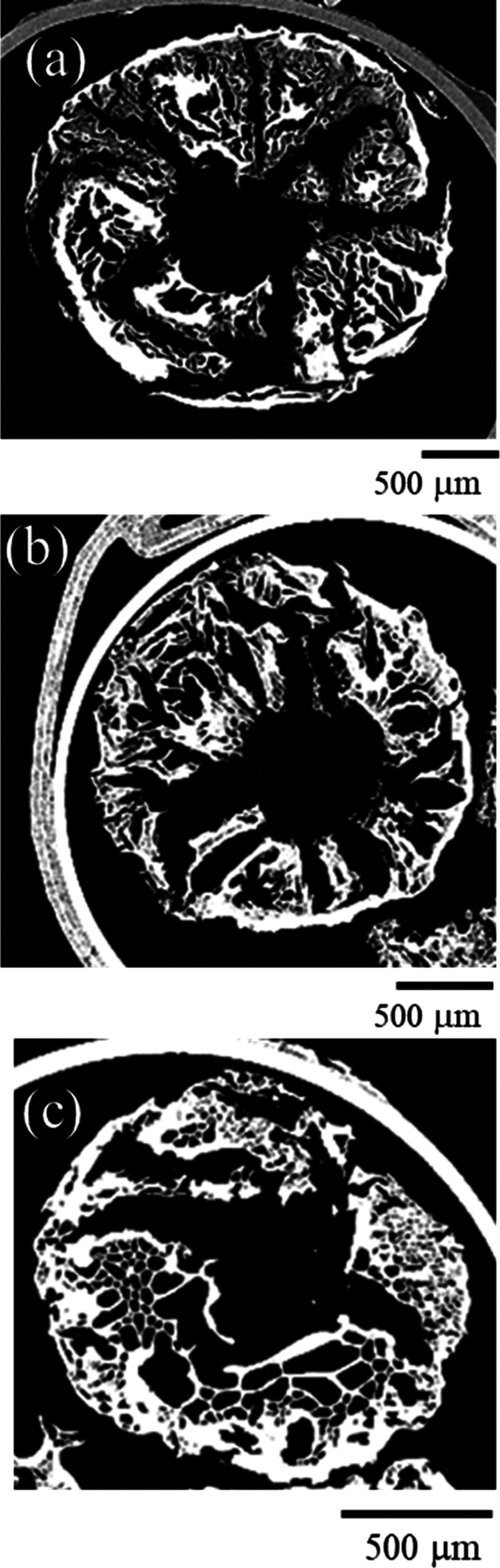
CT scan images of beads (a) 1.6 mm diameter needle, (b) 0.6 mm diameter needle, (c) 0.3 mm diameter needle.

3D rendered CT scans of the 0.6 mm beads are shown in Figure 5a, which show a relatively consistent morphology and surface texture, with some evidence of cracks on the surface. Figure 5b and c show a slice through the beads displaying a thin skin but also a hierarchical and accessible pore structure due to the cracks. Typical CT cross-sectional scans of one of each of the PIM-1 beads are presented in Figure 6.

The CT scans show similar internal structures with a clear network of different sized pores, where a large void in the center of the beads is clearly visible. This is likely due to the droplet freezing from the outside toward the center, where the initial freezing of the outer skin as it makes contact with the liquid nitrogen (see Figure 1) defines the dimensions of the bead. As the droplet continues to freeze, its total volume reduces during solidification, leaving the void in the center, since this would be the last region to freeze. The bead produced from the 1.6 mm needle (Figure 4a) has a larger proportion of small pores than the other beads. The bead produced from the 0.6 mm diameter needle (Figure 4b) has pores radiating from the hole in the center and more dense regions of material between. The bead produced from the smaller 0.3 mm diameter (Figure 4c) needle has a higher proportion of larger pores than the other beads. This could be because the size of the droplet is smaller and therefore freezes faster than the other beads, preventing the directional freezing from the outer shell to the center of the bead. Further work to CT scan the composite beads could aid in understanding the internal structure of composite beads, and how it differs from PIM-1 beads.

**Figure 7:**
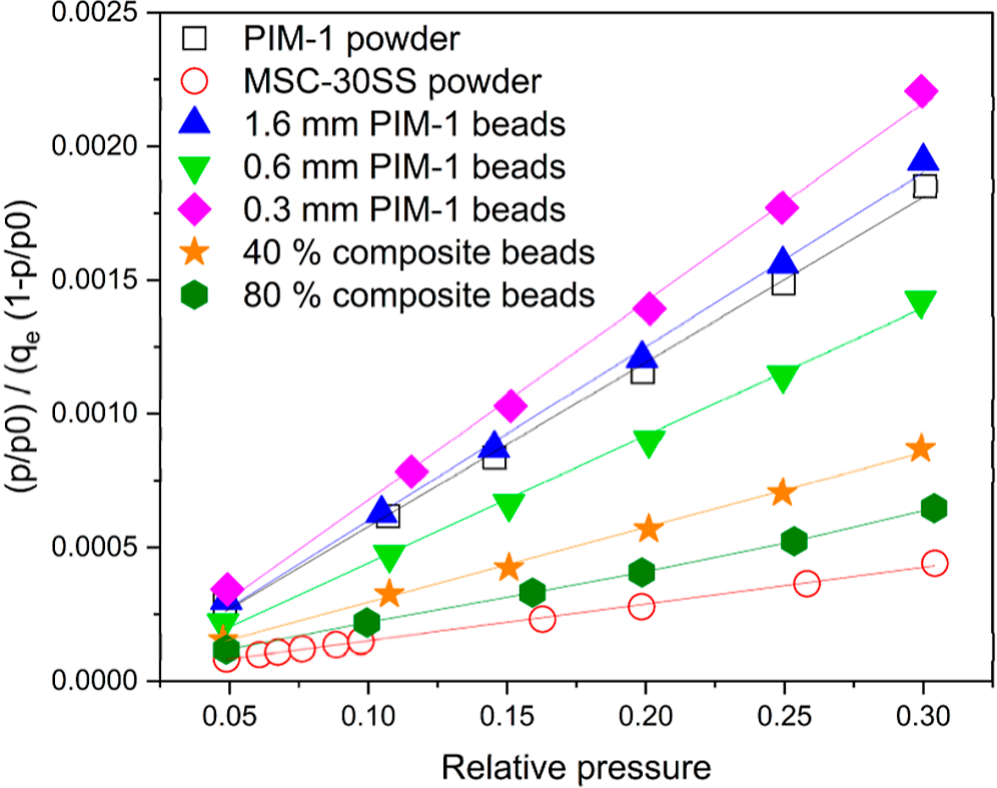
Linear form of the BET equation calculated from nitrogen isotherm data for PIM-1 powder, MSC-30SS powder, PIM-1 beads made with 1.6 mm diameter needle, 0.6 mm diameter needle, 0.3 mm diameter needle and PIM-1 MSC-30SS composite beads (40 and 80 wt %) made with 1.6 mm diameter needle. Symbols show isotherm data, lines are lines of best fit calculated using a linear fitting function in OriginPro 2024 software.

Nitrogen isotherms at 77 K were analyzed for the beads formed using the method set out in the experimental section. Data of the original PIM-1 and MSC-30SS powders are included for reference. The linear form of the BET equation (for *p*/*p*_0_ = 0.05–0.3^17^) for each of the powders and beads are presented in Figure 7, where hollow symbols represent the initial powdered materials (PIM-1 and MSC-30SS) and the filled symbols represent the beads. The ratio *p*/*p*_0_ (*y* axis) is relative pressure and *q*_e_ is the amount of gas adsorbed.

The MSC-30SS powder has the shallowest gradient in Figure 7, indicating a higher surface area, while the bead formed using the small 0.3 mm diameter needle has the steepest gradient indicating a lower surface area; this may be due to the more rapid freezing of the smaller droplets which limit the microporosity compared to the initial PIM-1 powder. The BET surface areas calculated are presented in Table 1. The beads formed from the 0.3 mm diameter needle have the lowest BET surface area of 584.1 m^2^ g^–1^, which shows a reduction in surface area compared to the PIM-1 powder, while retaining a reasonable surface area value. The MSC-30SS activated carbon powder has the highest BET surface area of 3068 m^2^ g^–1^. The BET surface areas for the PIM-1 40 wt % MSC-30SS composite beads follows a rule of mixtures (actual 1550 m^2^ g^–1^, predicted 1669 m^2^ g^–1^) given by eq 2
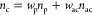
2where *n*_c_ is the surface area of the composite bead (m^2^ g^–1^), *w*_p_ is the weight fraction of polymer in composite bead, *n*_p_ is surface area of polymer powder (m^2^ g^–1^), *w*_ac_ is the weight fraction of activated carbon in the composite bead and *n*_ac_ is surface area of activated carbon powder (m^2^ g^–1^). However, for the 80 wt % MSC-30SS bead the predicted value is slightly higher than the actual value (actual 2167 m^2^ g^–1^, predicted 2602 m^2^ g^–1^).

**Table 1:** Nitrogen at 77 K BET Surface Areas for PIM-1 and MSC-30SS Powders and Beads

material	BET surface area (m^2^ g^–1^)
PIM-1 powder	737.5
MSC-30SS carbon powder	3068.4
PIM-1 bead: [1.6 mm diameter needle]	665.2
PIM-1 bead: [0.6 mm diam needle]	900.3
PIM-1 bead: [0.3 mm diam needle]	584.1
40% PIM-1 MSC-30SS bead: [1.6 mm diam needle]	1550.0
80% PIM-1 MSC-30SS bead: [1.6 mm diam needle]	2167.7

**Figure 8:**
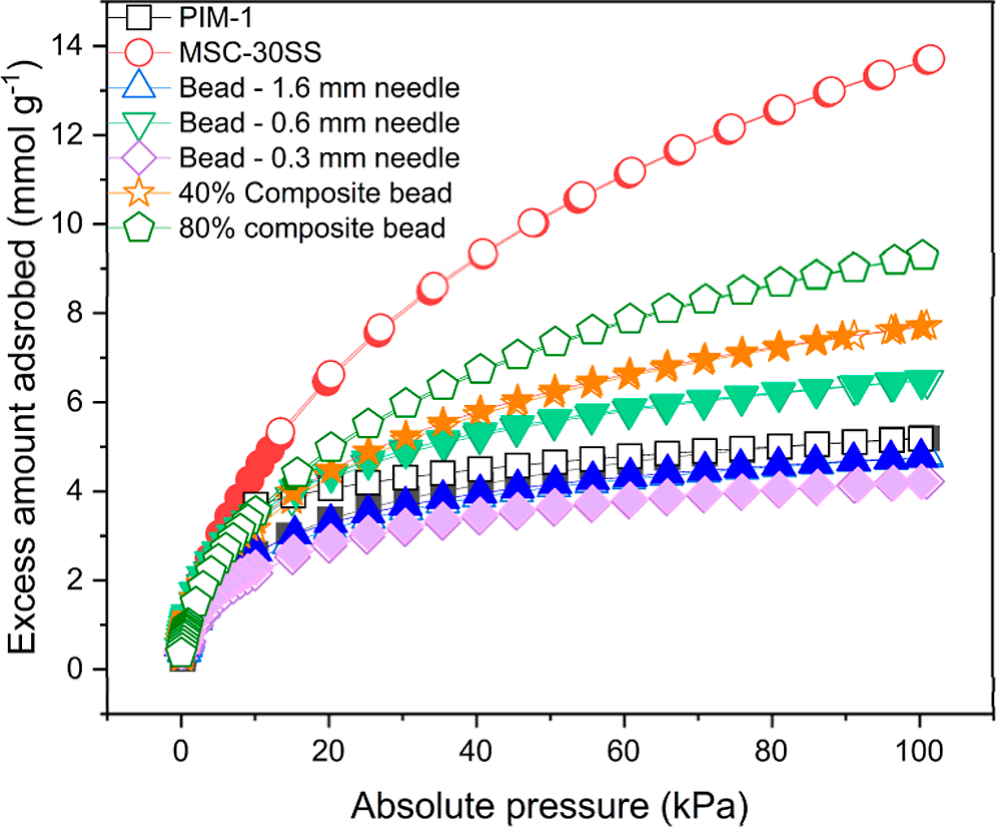
Hydrogen isotherm data for PIM-1 powder, MSC-30SS powder, PIM-1 beads made with 1.6 mm diameter needle, 0.6 mm diameter needle, 0.3 mm diameter needle and 40% and 80% composite beads made with 1.6 mm diameter needle. Filled symbols represent adsorption curves, hollow symbols represent desorption curves.

Hydrogen isotherms were produced for both the PIM-1 polymer powder and MSC-30SS carbon material, and the PIM-1 based beads formed. The resulting isotherms are presented in Figure 8. The hollow symbols represent the adsorption curves, and the filled symbols represent the desorption curves, where significant overlap occurs which indicates a fully reversible isotherm.

The MSC-30SS carbon powder adsorbed the most hydrogen (13.72 mmol g^–1^ at 100 kPa) since it has the largest surface area. The PIM-1 bead formed from the 0.3 mm diameter needle adsorbs the smallest amount of hydrogen (4.2 mmol g^–1^ at 100 kPa) since it has the smallest surface area (584.1 m^2^ g^–1^). The amount of hydrogen adsorbed by the 40 wt % MSC-30SS bead (7.7 mmol g^–1^) is close to values predicted by the rule of mixtures (8.6 mmol g^–1^). For the 80 wt % MSC-30SS bead the amount of hydrogen adsorbed (9.3 mmol g^–1^) is less than predicted by the rule of mixtures (11.9 mmol g^–1^); eq 2. This could be because the carbon powder is not homogeneously mixed when it is being fed through the needle or that some of the carbon pores have been blocked by the freeze casting process. Although the PIM-1 powder exhibits hysteresis (where the adsorption and desorption curves deviate from one another, Figure 8), this is not seen for the PIM-1 beads or composite beads, which is important for storage applications. This may be because macropores have been created in the structure during the freeze casting process or there is easier access to the pores that already exist in the material following freeze casting.

The experimental hydrogen adsorption data were fitted to the Tóth isotherm (eq 1) to predict the maximum storage capacity. Best fit parameters are presented in Table 2, along with the pressure that would be required to achieve 99% of the adsorption capacity, *q*_max_. The 0.3 mm bead has the lowest level of hydrogen storage (1.34 wt % at 428 kPa), with the MSC-30SS carbon exhibiting the highest (12.70 wt % at a pressure of 2.56 MPa). It should be noted that while these predicted pressures are approximately four times that used in the experiment, and they do provide a guideline on the achievable level of hydrogen storage. The predicted capacity of the individual powders and beads follows the same trend as the hydrogen storage at low pressure, namely that the largest capacity is for the MSC-30SS powder, the lowest capacity is for the 0.3 mm beads. The PIM-1 beads show similar predicted capacities to the PIM-1 powder and the 40 wt % MSC-30SS bead is similar to that predicted by the rule of mixtures. The MSC-30SS and 80 wt % MSC-30SS beads show promise to meet the Department of Energy (DoE) guidelines for light duty fuel cell vehicles as the wt % of hydrogen stored is significantly larger than that required (8.1 wt % although it is noted that the targets are for storage systems rather than storage materials). The level of hydrogen storage is higher than the other geometries reported to date (2.8 wt %,^5^ 7.1 wt %^21^). The predicted capacities and pressures calculated by fitting the experimental data to the Tóth equation have not been verified experimentally but the results for the activated carbon material (MSC-30SS) has been compared to literature values. Reference (4) shows that at 0.1 MPa the hydrogen uptake of AX21 is 2.5 wt % and at 10 MPa it is 9.24 wt %. For MSC-30SS the amount of hydrogen adsorbed is 13.72 mmol g^–1^, which is equivalent to 2.75 wt %. The fitting to the Tóth isotherm predicted that the capacity would be 12.2% at 2.5 MPa. The capacity of MSC-30SS is slightly more than that in ref 4, which is expected since the low pressure amount adsorbed is also slightly greater. The results for the MSC-30SS powder show good correlation, therefore, it is assumed that the Tóth isotherm is a suitable prediction for the capacity of the other powders and beads.

**Table 2:** Estimated Tóth Isotherm Parameters (See eq 1) for Hydrogen Adsorption at 77 K on Powders and Beads

material	*k* (kPa^–1^)	*t* (−)	*q*_max_ (wt %)	pressure to reach 99% *q*_max_ (kPa)
PIM-1 powder	0.207	0.447	1.75	477.96
MSC-30SS powder	0.039	0.331	12.20	2557.48
PIM-1 beads (0.3 mm needle)	0.231	0.459	1.34	428.57
PIM-1 beads (0.6 mm) needle	0.238	0.452	2.09	415.97
PIM-1 beads (1.6 mm needle)	0.227	0.466	1.50	436.12
composite beads (40 wt % carbon)	0.115	0.357	4.12	860.87
composite beads (80 wt % carbon)	0.09	0.29	8.1	1100

When compared to research to date, films of PIM-1 cast from solution were produced by Tian et al.^21^ However, the surface area reduced to 600 m^2^ g^–1^ compared to 724 m^2^ g^–1^ for the PIM-1 powder. Composite films of PIM-1 doped with “filler” materials have also been produced to improve surface area. Tian et al.^21^ produced composite membranes by evaporating solutions of PIM-1 dissolved in chloroform with a highly microporous composite filler, AX21, which has a surface area of 2975 m^2^ g^–1^. A weight fraction of 60 wt % AX21 was the maximum amount of filler that could be added for a successful film to be developed and showed a surface area of 2034 m^2^ g^–1^, which follows the rule of mixtures according to wt % of materials present. The hydrogen uptake of the films was found to be proportional to the amount of filler that is added.^21^ The films demonstrated hydrogen storage capacities of up to 3 wt % with predicted limiting values up to 7 wt %.^21^ These films had a lower surface area (<2034 m^2^ g^–1^) and hydrogen storage capacity (<7 wt %) than the composite beads produced here (4.1 wt %, 8.1 wt %). Alongside this, the maximum amount of filler that could be added to the films reported by Tian et al.^21^ was limited to 60 wt %; however, for the beads produced here, up to 80 wt % carbon was added. Higher percentages were not investigated but there is potential to improve this further. Films are also limited by thickness and repeatability whereas beads would have better repeatability and potential to be reproduced at scale.

Our focus here is to demonstrate a simple process to produce beads for hydrogen storage applications, and provide initial indicators of sorption capacity of the beads via BET analysis and hydrogen storage capacity. We recognize that pore size distribution and pore volume are important parameters for hydrogen storage. In future work, these quantities can be analyzed for both the powders and beads to further understand the effect of forming on these parameters on sorption capacity.

## Conclusions

This paper provides the first successful demonstration of the use of droplet freeze casting to form beads of an adsorbent polymer of intrinsic microporosity (PIM-1) and composite beads based on a PIM-1 matrix that is filled with activated carbon. The surface areas of the beads remain consistent compared to the raw powders and the 40 wt % MSC-30SS beads follow the rule of mixtures, enabling prediction and design of bead properties for storage. At higher percentages of fillers the surface area and hydrogen storage capacity fall slightly (16%) below that predicted by the rule of mixture. The intriguing mixture of microporosity (within the PIM-1 matrix or active carbon filler) and the macropores (which are introduced by the freeze casting process) has potential to provide a combination of gas storage and high mass transfer. The mass of hydrogen stored in PIM-1 beads differs slightly from the PIM-1 powder, where the composite beads are predicted to reach 8.1 wt %, which has potential to meet the Department of Energy (DoE) targets for light duty fuel cell vehicles. This work therefore represents the first example of producing PIM-based beads for gas storage or separation applications. In addition, the level of hydrogen storage is higher than the other geometries reported to date (2.8 wt %,^5^ 7.1 wt %^21^).

This relatively simple, saleable and easily tailored process provides a range of opportunities for further investigation. The next stage of development would be to understand composite bead production in more detail in particular the optimal flow rate for pipetting the composite solution into the liquid nitrogen should be investigated due to the differing viscosity of the solution as a result of adding the filler material. This would enable a smaller variation in the size of the beads formed and using a higher flow rate would limit the amount of time the solution had to settle or separate. Beads could potentially be formed successfully with higher wt % of filler material. In addition additional filler material could be investigated and lead to improved surface area and hydrogen storage capacity for beads. PIM-1 based composite beads show great potential for hydrogen storage applications and many areas for further investigation and viable improvements.

## Abbreviations

PIM-1: polymer of intrinsic microporosity 1
BET: Brunauer Emmet Teller
DoE: Department of Energy
COF: covalent organic frameworks
PAF: porous aromatic frameworks
MOFs: metal organic frameworks
AC: activated carbons
KOH: potassium hydroxide
K_2_CO_3_: anhydrous potassium carbonate
DMF: dimethylformamide
CT: computed tomography
p: absolute pressure
*p*_0_: saturation pressure
*q*: the amount of gas adsorbed
nc: the surface area of the composite bead (m^2^ g^–1^)
wt % p: percentage weight of polymer in composite bead
np: surface area of polymer powder (m^2^ g^–1^)
nac: surface area of activated carbon powder (m^2^ g^–1^)
